# Identification of biomarkers related to lymph node metastasis and prognosis in thyroid cancer based on integrated bioinformatics exploration

**DOI:** 10.1016/j.clinsp.2026.101201

**Published:** 2026-10-15

**Authors:** Xuejian Hu, Dan Ge, Xiaolan Ma, Yan Yang

**Affiliations:** Department of Endocrinology and Metabolism, The Second Hospital & Clinical Medical School, Lanzhou University, Lanzhou, Gansu, China

**Keywords:** Thyroid neoplasms, Lymphatic metastasis, Biomarkers, Immune infiltration, Computational biology

## Abstract

**Objective:**

Thyroid Carcinoma (THCA) is a common endocrine malignancy, and Lymph Node Metastasis (LNM) is a critical determinant of poor prognosis. This study aimed to identify LNM-related biomarkers and establish a prognostic model for THCA patients using integrated bioinformatics approaches.

**Methods:**

Clinical data from SEER were analyzed to identify prognostic factors in THCA. Transcriptomic data from TCGA were used to identify differentially expressed genes between LNM and non-LNM samples. Survival analysis, machine learning (Boruta and LASSO), and functional enrichment were applied to screen candidate biomarkers. Immune infiltration and a ceRNA network were also explored.

**Results:**

Six independent prognostic factors were identified from SEER (n = 38,955): age, gender, marital status, race, pathologic T-stage, and pathologic M-stage. A nomogram incorporating these factors showed good predictive performance (AUC = 0.744). Transcriptomic analysis revealed 332 LNM-related genes. Through integrative machine learning, six biomarkers ‒ CLDN10, OTOS, NEXMIF, ACOT6, ASXL3, and SLC4A4 ‒ were selected. These genes were predominantly enriched in immune response and metabolic pathways. M0 macrophages and resting dendritic cells showed significant abundance differences between LNM and NLNM samples, with CLDN10 positively correlated with resting dendritic cells (R = 0.47, adjusted p < 0.01). A ceRNA network involving 14 miRNAs and 4 lncRNAs was constructed, with hsa-miR-15a-5p predicted to regulate SLC4A4, ASXL3, and NEXMIF.

**Conclusion:**

We identified six candidate LNM-related biomarkers and clinical factors associated with THCA prognosis. These findings provide a basis for further investigation into LNM mechanisms and may offer new perspectives for prognostic assessment, though independent validation is required.

## Highlights

• We detected significant difference of overall survival time between LNM and Non-LNM (NLNM) patients.
• Six LNM-related biomarkers, *CLDN10, OTOS, NEXMIF, ACOT6, ASXL3* and *SLC4A4*, were identified by analyzing transcriptomic data from the TCGA-THCA database.
• Immune cells such as M0 macrophages and resting dendritic cells showed significant differences between LNM and NLNM samples.
• Hsa-miR-15a-5p was found to potentially regulate *SLC4A4, ASXL3* and *NEXMIF* by competitive endogenous RNA (ceRNA) prediction.

## Introduction

Thyroid Carcinoma (THCA) is a malignant tumor originating from the follicular epithelium or parafollicular epithelial cells of the thyroid gland.^1^^,^^2^ Thyroid gland, the largest endocrine gland in the human body, is located below the thyroid cartilage in the neck, on both sides of the trachea. It synthesizes, stores, and secretes thyroid hormones, playing a crucial regulatory role in metabolism, growth, and development. Dysfunction of thyroid gland can lead to various diseases, including THCA, the pathogenesis of which involves multiple factors. From a molecular biology perspective, the genetic and epigenetic alterations of certain genes in several major signaling pathways are key factors in the development of THCA.^3^^,^^4^ For example, RET/PTC gene rearrangement is closely related to THCA, and can lead to abnormal cell proliferation and differentiation.^5^ At cellular level, normal thyroid cells lose control of their growth and division when they are stimulated by various carcinogenic factors, gradually forming cancer cells.^6^ Base on this understanding, effective biomarkers identification is of great significance for early diagnosis, treatment, and prognostic prediction in THCA.

Lymph Node Metastasis (LNM), the spread of thyroid cancer cells from the primary lesion to adjacent lymph nodes, is one of the most common metastatic routes in THCA.^7^ LNM is more common in cases of significant local progression or when the tumor exceeds 1 cm in size.^8^ Once LNM is detected, lymph node dissection is typically required to ensure complete removal of residual cancer cells. In addition, LNM influences tumor staging and prognostic evaluation.^9^ For THCA patients, regular neck ultrasound, nuclear scanning, and lymph node biopsy are essential for early LNM detection and timely intervention.^10^ It has been reported that seven genes are associated with lymphatic metastasis and also linked to tumor immune cell infiltration.^11^ Therefore, it is necessary and important to explore biomarkers related to LNM to predict the overall prognosis of THCA patients.

In this study, we systematically investigated the biomarkers associated with LNM of THCA patients based on their clinical information from the Surveillance, Epidemiology, and End Results (SEER) database and transcriptome data (RNA-seq) of THCA patients from the Cancer Genome Atlas (TCGA) database. Using bioinformatics methods, a prognostic model was constructed to explore the efficiency of biomarkers related to LNM of THCA, providing new references for the clinical diagnosis, treatment, and prognosis of THCA patients.

## Materials and methods

### Data source of this study

The THCA RNA-seq dataset was obtained from The Cancer Genome Atlas (TCGA) database (http://cancergenome.nih.gov/), comprising 513 THCA tumor samples and 59 normal control samples. Survival datasets of THCA patients with complete survival time and survival status, including 408 THCA samples, were obtained from University of California Santa Cruz (UCSC) xena database (https://xenabrowser.net/datapages/). After excluding samples with unavailable or ambiguous lymph node status (NX or missing N-stage) and samples lacking complete survival data, the reminding TCGA-THCA samples were categorized into LNM (n = 204) and Non-LNM (NLNM) (n = 204) groups. THCA samples from the Surveillance, Epidemiology, and End Results (SEER) database (https://seer.cancer.gov/) were also included in this study. The primary site of thyroid gland was labeled by C73.9. Likewise, SEER-THCA samples were categorized into LNM samples and NLNM samples.

### Construction of a nomogram for predicting the risk of LNM

The distribution of clinical characteristics (age, gender, vital status, Overall Survival [OS], pathologic TNM stage, marital status, race, laterality) was compared between LNM and NLNM patients in SEER-THCA dataset using chi-square test (n = 38,955, LNM = 9800, NLNM = 29,155). Survival analysis was executed to evaluate OS and Cancer-Specific Survival (CSS) between LNM and NLNM patients by survminer package (v 0.4.9).^12^ Afterwards, a nomogram analysis was constructed by rms package (v 6.5–0)^13^ to assess the clinical risk of LNM in THCA patients, incorporating age, gender, pathologic TM stage, marital status, race, and laterality. Receiver Operating Characteristic (ROC) analysis was performed to calculate the Area Under the Curve (AUC) in order to assess the predictive performance of nomogram. Calibration curves were used to evaluate the association between actual probability and predicted probability. Additionally, Decision Curve Analysis (DCA) was applied by rmda package (v 1.6) to assess whether nomogram had a superior ability in risk prediction compared to using age, gender, pathologic TM stage, marital status, race, or laterality alone. And a clinical impact curve was constructed via DCA curve to visualize clinical implications of nomogram.

### Independent prognostic analysis in SEER-THCA dataset

Based on SEER-THCA dataset, we identified independent prognostic factors for OS (n = 9800) and CSS (n = 9109) of THCA patients with LNM. First, univariate Cox regression analysis was performed to evaluate the prognostic significance of clinical characteristics (gender, age, marital status, race, laterality, pathologic TM stage) by survival package (v 3.3–1)^14^ (p < 0.05). Then, independent prognostic factors were obtained by multivariate Cox analysis (p < 0.05) of variables that passed the proportional hazards hypothesis test. Next, KM survival analysis was conducted to evaluate OS and CSS by independent prognostic factors of different clinical subtypes using survminer package. Next, cumulative incidence analysis of independent prognostic factors was conducted using cmprsk (v 2.2–11) package.^15^ Furthermore, we developed a nomogram to predict OS of THCA patients at 1-, 3-, and 5-years using the identified independent prognostic factors. The predictive survival capability of nomogram was evaluated using calibration curves and ROC curves. DCA was used to evaluate the effectiveness of nomogram as an optimal model. Thereafter, THCA patients were dichotomized into high and low risk groups by median value of nomogram score. Finally, survival differences from both groups were assessed.

### Identification and enrichment analysis of LNM-related genes

In TCGA-THCA dataset, Differentially Expressed Genes (DEGs) were obtained between cancer and control samples (n = 572, cancer = 513, control = 59) [|log_2_Fold Change (FC)| > 1 and adj.p < 0.05] by DESeq2 package (v 1.36.0),^16^ denoted as THCA-DEGs. DEGs were also identified between LNM and NLNM samples (n = 408, LNM = 204, NLNM = 204), denoted as metastases-DEGs. Volcano plot and heatmap of DEGs were presented by ggplot2 package (v 3.4.0)^17^ and ComplexHeatmap (v 2.12.1),^18^ respectively. Then, LNM-related genes were obtained by overlapping THCA-DEGs and metastases-DEGs. GO and KEGG enrichment analysis (p < 0.05) of metastases-DEGs were analyzed by ClusterProfiler package (v 4.4.4)^19^ and org.Hs.eg.db package (v 3.15.0).^20^ The enriched GO pathways were visualized via GOplot package (v 1.0.2).^21^ To explore the interactions among LNM-related genes at protein level, LNM-related genes were uploaded to the online STRING database (https://cn.string-db.org/) for Protein-Protein Interaction (PPI) network construction (confidence value = 0.7). The visualization of this network was achieved by Cytoscape software (v 3.8.2).^22^

### Identification and expression analysis of LNM-related biomarkers

Initially, LNM-related genes were extracted and integrated with the survival data. We then categorized TCGA-THCA samples into high and low expression groups according to the median expression value of each gene. Next, we compared survival differences between high and low expression groups and identified genes significantly correlated with survival of THCA patient (p < 0.05). Then, Boruta algorithm and Least Absolute Selection and Shrinkage Operator (LASSO) Cox regression analysis were conducted to further acquire LNM-related biomarkers using Boruta package (v 7.0.0)^23^ and glmnet package (v 4.1–6),^24^ respectively. Ultimately, survival curves for LNM-related biomarkers were plotted, and the expression levels of LNM-related biomarkers in TCGA-THCA samples were analyzed using ggpubr package (v 0.4.0). Finally, the expression levels of LNM-related biomarkers were validated by GEPIA database.

### Functional and annotation analyses

In order to gain insights into biological functions and pathways associated with LNM-related biomarkers, Gene Set Enrichment Analysis (GSEA) was conducted for the correlated genes with each LNM-related biomarker by ClusterProfiler package (p < 0.05). The results were visualized using GseaVis (v 0.0.5), with the Hallmark gene set serving as background.

### Immune infiltration analysis

To further assess immune cell composition, change in immune microenvironment of THCA patients, the proportions of 22 immune cells were calculated in TCGA-THCA samples using the CIBERSORT algorithm,^25^ and differences in immune cell proportions between THCA and control samples as well as between LNM and NLNM samples were compared (p < 0.05). In addition, Spearman correlation analysis was conducted between immune cells and LNM-related candidate biomarkers with false discovery rate (adjusted p-value) < 0.05 as significant.

### Construction of regulatory network

In order to explore molecular regulatory mechanisms of LNM-related biomarkers in THCA, the miRNAs interacting with LNM-related biomarkers were predicted by miRande database, miRDB database, and TargetScan database by multiMiR package (v 1.18.0).^26^ Then we obtained the intersection of the predicted miRNA across all three databases. Besides, lncRNAs bound by the intersected miRNAs were predicted using starBase database (geneType = lncRNA, clipExpNum ≥ 7 and pancancerNum ≥ 4). Eventually, a competitive endogenous RNA (ceRNA) network was established and visualized using Cytoscape software.

### Statistical analysis

All analyses were executed in R language (v 4.2.3). Differences between groups were analyzed by Wilcoxon test; p < 0.05 was considered as statistically significant.

## Results

### LNM patients survived shorter than NLNM patients

First, we observed significant differences between LNM and NLNM patients in all clinical subtypes (p < 0.001) (Table 1). By performing Overall Survival (OS) and Cancer Specific Survival (CSS) analysis, we found that LNM patients had significantly shorter survival than NLNM patients (p < 0.0001) (Fig. 1A‒B). Therefore, we constructed a nomogram to identify clinical risk factors of LNM (Fig. 1C). The calibration curve and ROC curve (AUC = 0.744) demonstrated a favorable accuracy in predicting the risk of LNM patients by combining 7 clinical factors: age, gender, two pathologic TM stages, marital status, race, and laterality (Fig. 1D). DCA demonstrated that the nomogram provided greater ability to predict risk compared to the usage of age, gender, pathologic TM stage, marital status, race, or laterality alone (Fig. 1E). The clinical impact curve showed a close overlap between “Number of high-risk” curve and “Number of high-risk with event” curve, indicating that nomogram exhibited superior predictive accuracy (Fig. 1F). In summary, these results demonstrate clear clinical difference between LNM and NLNM patients.

**Table 1:** Clinical data difference between LNM and NLNM samples in SEER.

**Clinical features**	**Total**	**LN metastases**	**No metastases**	**p-value**
**Age (year)**				
Mean (SD)	49.4 (±16.1)	46.4 (±16.6)	50.4 (±15.7)	<0.001
**gender**				
Female	28,383 (72.9%)	6470 (66.0%)	21,913 (75.2%)	<0.001
Male	10,572 (27.1%)	3330 (34.0%)	7242 (24.8%)	
**Vital status**				
Alive	35,507 (91.1%)	8258 (84.3%)	27,249 (93.5%)	<0.001
Dead	3448 (8.9%)	1542 (15.7%)	1906 (6.5%)	
**OS (months)**				
Mean (SD)	70.3 (±55.8)	103.9 (±50.0)	59.0 (±53.0)	<0.001
**Pathologic_M**				
M0	19,215 (95.9%)	9051 (93.4%)	10,164 (98.4%)	<0.001
M1	582 (2.9%)	468 (4.8%)	114 (1.1%)	
MX	231 (1.2%)	176 (1.8%)	55 (0.5%)	
**Pathologic_N**				
N0	10,333 (51.6%)	0 (0.0%)	10,333 (100.0%)	<0.001
N1	9695 (48.4%)	9695 (100.0%)	0 (0.0%)	
**Pathologic_T**				
T0	67 (0.3%)	62 (0.6%)	5 (0.0%)	<0.001
T1	10,051 (50.2%)	3483 (35.9%)	6568 (63.6%)	
T2	3303 (16.5%)	1565 (16.1%)	1738 (16.8%)	
T3	4774 (23.8%)	3274 (33.8%)	1500 (14.5%)	
T4	1261 (6.3%)	1025 (10.6%)	236 (2.3%)	
TX	572 (2.9%)	286 (2.9%)	286 (2.8%)	
**Marital status**				
Divorced	2649 (6.8%)	598 (6.1%)	2051 (7.0%)	<0.001
Married (including common law)	23,221 (59.6%)	5816 (59.3%)	17,405 (59.7%)	
Separated	293 (0.8%)	58 (0.6%)	235 (0.8%)	
Single (never married)	8727 (22.4%)	2505 (25.6%)	6222 (21.3%)	
Unknown	2109 (5.4%)	395 (4.0%)	1714 (5.9%)	
Unmarried or Domestic Partner	212 (0.5%)	35 (0.4%)	177 (0.6%)	
Widowed	1744 (4.5%)	393 (4.0%)	1351 (4.6%)	
**Race**				
Black	1961 (5.0%)	275 (2.8%)	1686 (5.8%)	<0.001
Other (American Indian/ AK Native, Asian/ Pacific Islander)	6121 (15.7%)	1504 (15.3%)	4617 (15.8%)	
Unknown	662 (1.7%)	108 (1.1%)	554 (1.9%)	
White	30,211 (77.6%)	7913 (80.7%)	22,298 (76.5%)	
**Laterality**				
Bilateral, single primary	314 (0.8%)	43 (0.4%)	271 (0.9%)	<0.001
Left - origin of primary	1025 (2.6%)	116 (1.2%)	909 (3.1%)	
Not a paired site	36,291 (93.2%)	9493 (96.9%)	26,798 (91.9%)	
Only one side ‒ side unspecified	2 (0.0%)	0 (0.0%)	2 (0.0%)	
Paired site, but no information concerning laterality	16 (0.0%)	2 (0.0%)	14 (0.0%)	
Right ‒ origin of primary	1307 (3.4%)	146 (1.5%)	1161 (4.0%)

**Fig. 1:**
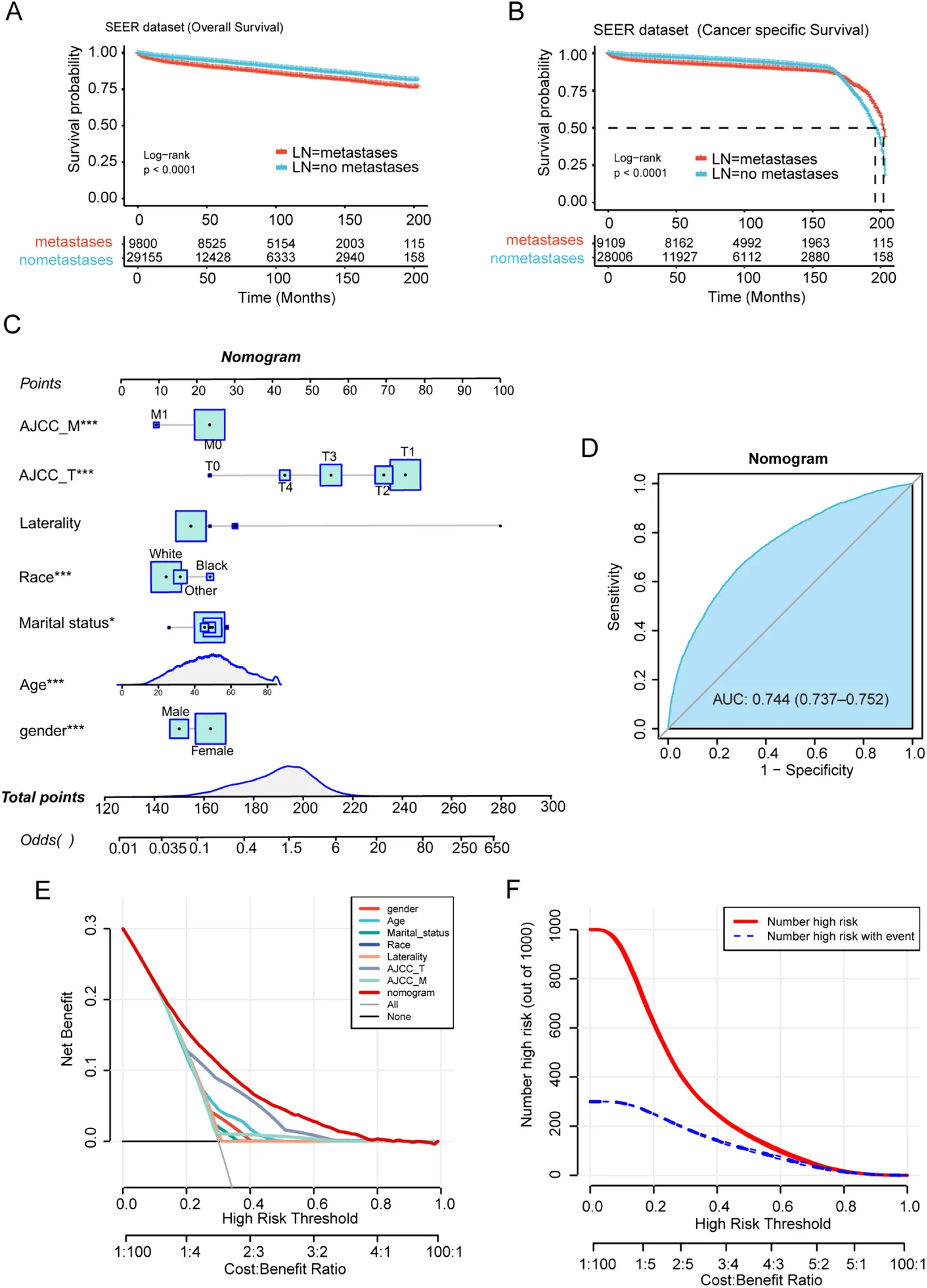
Survival analysis and the difference between LNM and NLNM patients. (A) The OS curve for LNM and NLNM patients. (B) The CSS curve for LNM and NLNM patients. (C) Nomogram analysis result for the seven risk factors. The * point represented significant difference. (D) ROC curve for evaluating the predictive ability of nomogram analysis model. (E) DCA result for the combined factors and individual factors. (F) The clinical impact curve analysis between “Number of high-risk” curve and “Number of high-risk with event” curve.

### Age, gender, marital status, race, pathologic T-stage, pathologic M-stage were independent factors for prognosis of THCA patients

To identify the influential factors for OS of THCA patients, we performed Cox regression analysis and found that gender, age, marital status, race, laterality, pathologic T-stage, and pathologic M-stage were significantly associated with OS of THCA patients (p < 0.05 and HR≠1) (Fig. 2A). Then, we performed pH hypothesis test to incorporate variables that were satisfied with the proportional hazard assumption into the subsequent analyses. Ultimately, we identified that age, gender, marital status, race, pathologic T-stage, and pathologic M-stage were independent factors that can predict the prognosis of THCA patients (p < 0.05 and HR≠1) (Fig. 2B). Notably, our independent prognostic analysis of CSS yielded identical results. Moreover, patients with age > 50, male, widowed, black, T4-stage, and M1 stage experienced a significantly worse OS (p < 0.0001) (Fig. 2C‒H). In summary, we identified a set of robust clinical factors for predicting survival in THCA patients that can be used as survival predictor.

**Fig. 2:**
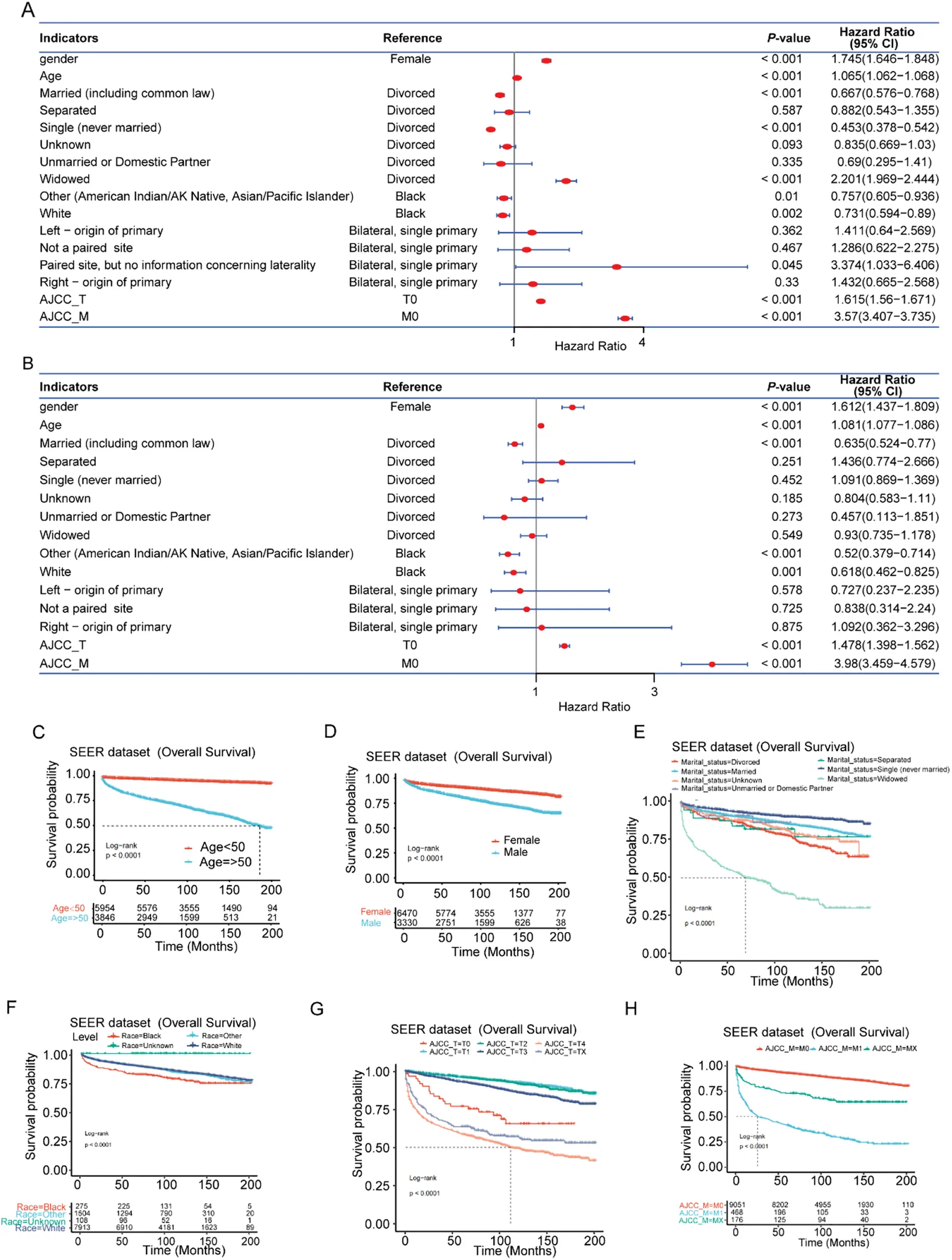
Prognostic factor analysis for THCA patients. (A‒B) Single factor (A) and multivariate (B) Cox independent prognostic analysis of OS for LNM patients. (C‒H) K-M analysis of the six independent prognostic factors in patients with LNM.

### Construction of nomogram of OS and CSS

Consequently, we developed a nomogram incorporating the identified independent prognostic factors of OS to predict 1-, 3-, and 5-year survival rates of THCA patients (Fig. 3A). Both models demonstrated strong predictive performance, as evidenced by well-calibrated curves showing high concordance with the reference line and ROC curves with AUC values exceeding 0.9 at all-time points (Fig. 3B‒C). Obviously, DCA curves further confirmed the clinical utility of the nomograms, showing superior net benefit compared with individual prognostic factor (Fig. 3D‒H). Furthermore, using the median nomogram score, we stratified patients into high- and low-risk groups, revealing significantly poorer OS and CSS in the high-risk cohort (p < 0.0001) (Fig. 3I‒J). Collectively, these findings indicate that the nomograms integrating age, gender, marital status, race, pathologic T-stage, and pathologic M stage represent robust tools with significant potential for clinical application in THCA patient management.

**Fig. 3:**
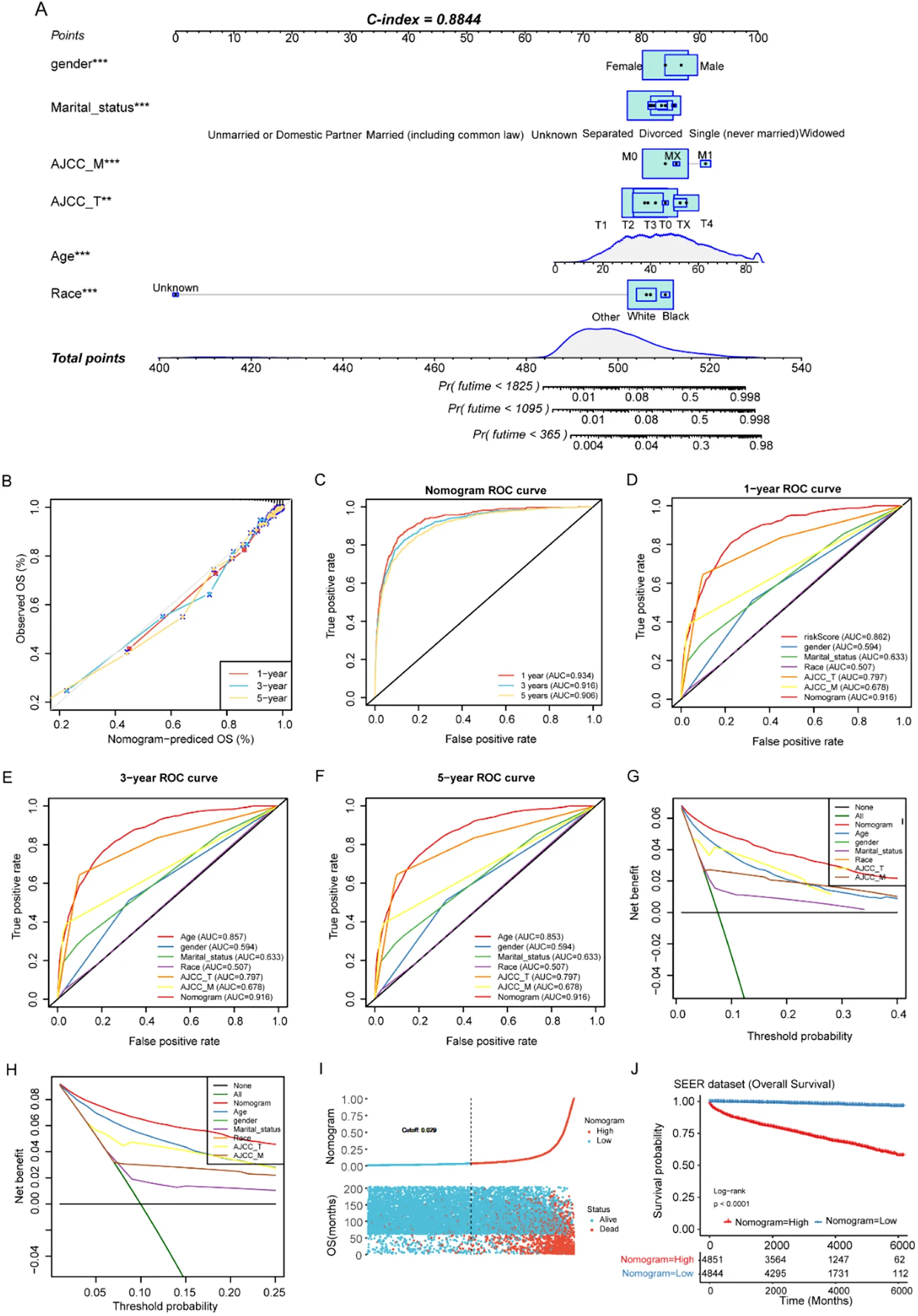
Construction and analysis results of OS nomogram. (A) Nomogram analysis result for the six prognostic factors. (B) Correction curve for predicting 1-, 3-, and 5-year survival rates of THCA patients. (C) ROC curve for predicting 1-, 3-, and 5-year survival rates of THCA patients. (D‒F) ROC curve for predicting 1-, 3-, and 5-year survival rates for the six prognostic factors of THCA patients. (G‒H) DCA result for the combined factors and individual factors. (I) The risk curve and scatter plot. (J) The survival curve for the nomogram high- and low-risk patients.

### Transcriptome analysis to identify LNM-related genes

Our SEER-THCA dataset analysis revealed a strong correlation between LNM and poor prognosis of THCA patients, implicating a critical determinant of LNM in the metastasis of THCA. To identify molecular biomarkers linking LNM to THCA metastasis, we conducted further investigations on TCGA-THCA transcriptome dataset. A total of 3021 THCA-DEGs were identified, including 1811 up-regulated and 1210 down-regulated genes, between THCA tumor and control samples from TCGA-THCA dataset (Fig. 4A‒B). In the same way, analysis of LNM vs. NLNM samples yielded 533 metastasis DEGs (180 upregulated, 353 downregulated) (Fig. 4C‒D). And in total 332 LNM-related genes were obtained by intersecting THCA-DEGs and metastases-DEGs (Fig. 4E). In order to further explore the relevant signaling pathways and potential biological mechanisms underlying the LNM of THCA, LNM-related genes were incorporated into GO and KEGG analysis. Concretely, they were primarily associated with “keratinization”, “skin development”, and “extracellular matrix organization” in GO-BP pathways (Fig. 4F), and were enriched in “IL-17 signaling pathway”, “Neuroactive ligand-receptor interaction”, “Hematopoietic cell lineage” and other KEGG pathways (Fig. 4G). These analyses revealed the importance of these signaling pathways in the metastasis of THCA. Furthermore, by integrating 332 LNM-related genes with confidence value > 0.7, 117 nodes and 150 edges were identified, with *NMU* serving as a hub gene connected to *GPR83, NPY, PPY* and *MLN* (Fig. 4J).

**Fig. 4:**
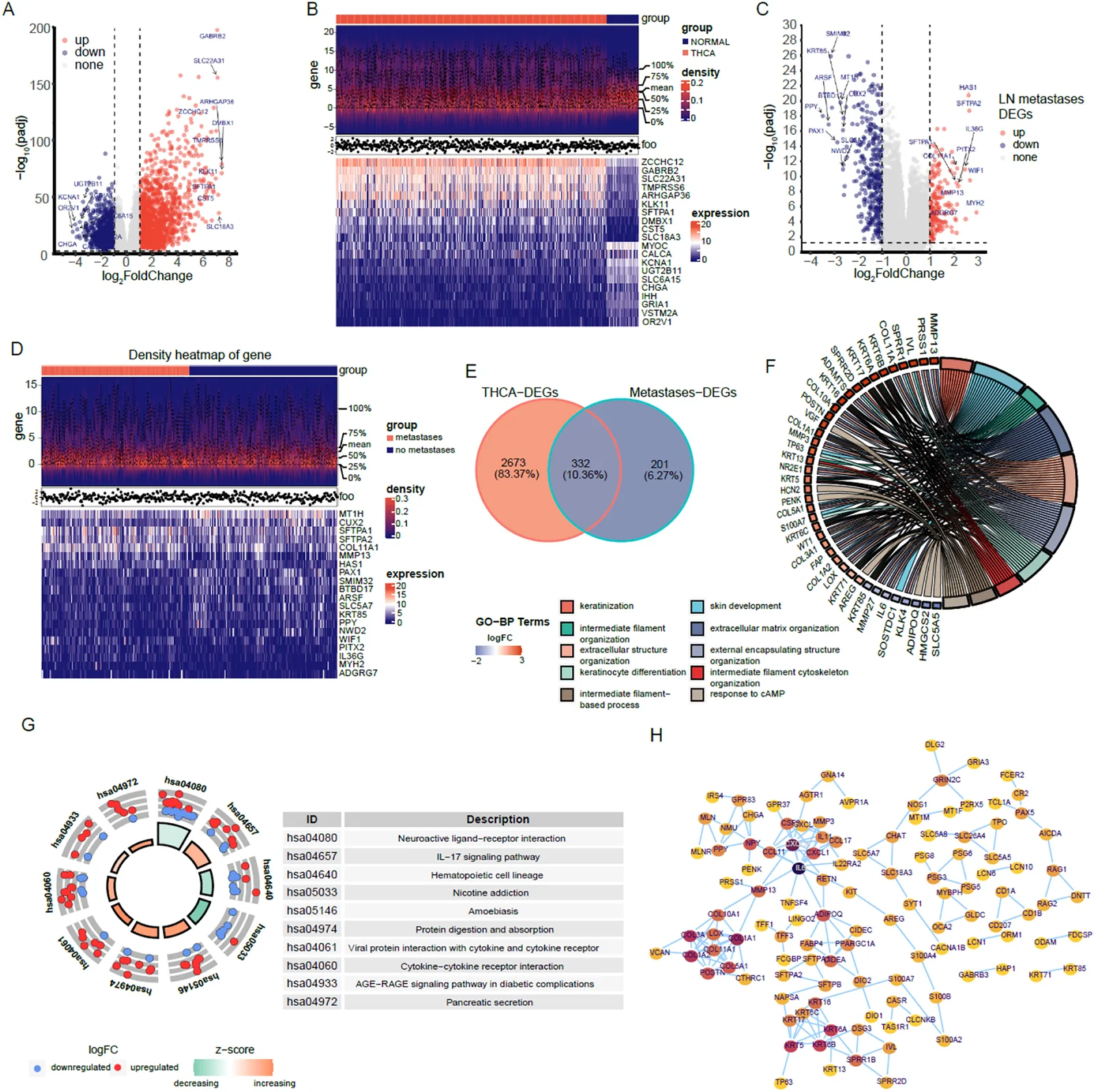
Expression analysis of LNM-related DEGs from TCGA dataset. (A) Volcano plot showing the DEGs from TCGA-THCA dataset. The symbols of top DEGs were labeled in this figure. (B) Clustering heatmap showing the top DEGs and their expression density. (C) The same as (A) but for DEGs between LNM and NLNM samples. (D) The same as (B) but for the top DEGs between LNM and NLNM samples. (E) Venn diagram of the overlapped DEGs between THCA-DEGs and LNM-DEGs. (F‒G) GO-BP (F) and KEGG (G) enrichment results for LNM-associated DEGs. (H) PPI network presentation for LNM-associated DEGs.

### CLDN10, OTOS, NEXMIF, ACOT6, ASXL3 and SLC4A4 were identified as LNM-related biomarkers

To further identify robust LNM-related biomarkers, we performed survival analysis on the 332 LNM-associated genes, revealing 26 genes significantly correlated with the survival of THCA patients (Supplementary Table 1). Machine learning-based feature selection was then applied to further screened out LNM-related biomarkers. The Boruta algorithm identified 16 candidate genes, including *CLDN10, OTOS*, and *NEXMIF* (Supplementary Table 2 and Fig. 5A), while LASSO Cox regression analysis identified 8-genes, including *CIDEC, CLDN10, OTOS, NEXMIF, INSM2, ACOT6, ASXL3*, and *SLC4A4* (Fig. 5B). Finally, we identified 6-genes by overlapping both machine learning algorithm results, namely *CLDN10, OTOS, NEXMIF, ACOT6, ASXL3* and *SLC4A4*, defined as LNM-related potential candidate biomarkers (Fig. 5C)*.* The survival analysis demonstrated that high *CLDN10* expression group exhibited a significantly better OS compared to low expression groups (p < 0.05), whereas high expression of *OTOS, NEXMIF, ACOT6, ASXL3* predicted poorer OS (all p < 0.05) (Fig. 5D). We next evaluated the expression patterns of these six LNM-related candidate biomarkers in both TCGA-THCA dataset and GEPIA database. Explicitly, *CLDN10* showed higher expression level, while *OTOS, NEXMIF, ACOT6, ASXL3* and *SLC4A4* showed lower expression levels in THCA samples compared to normal samples (p < 0.0001) (Fig. 5E). The same expression trends were obtained and validated by the GEPIA database (Fig. 5F). We observed discordance between expression levels and OS results for the candidate biomarkers; CLDN10 may be a marker of differentiated, less aggressive tumors, whereas the other five genes, though downregulated in tumors, may promote aggressive behavior when expressed above a certain threshold within the tumor population.

**Fig. 5:**
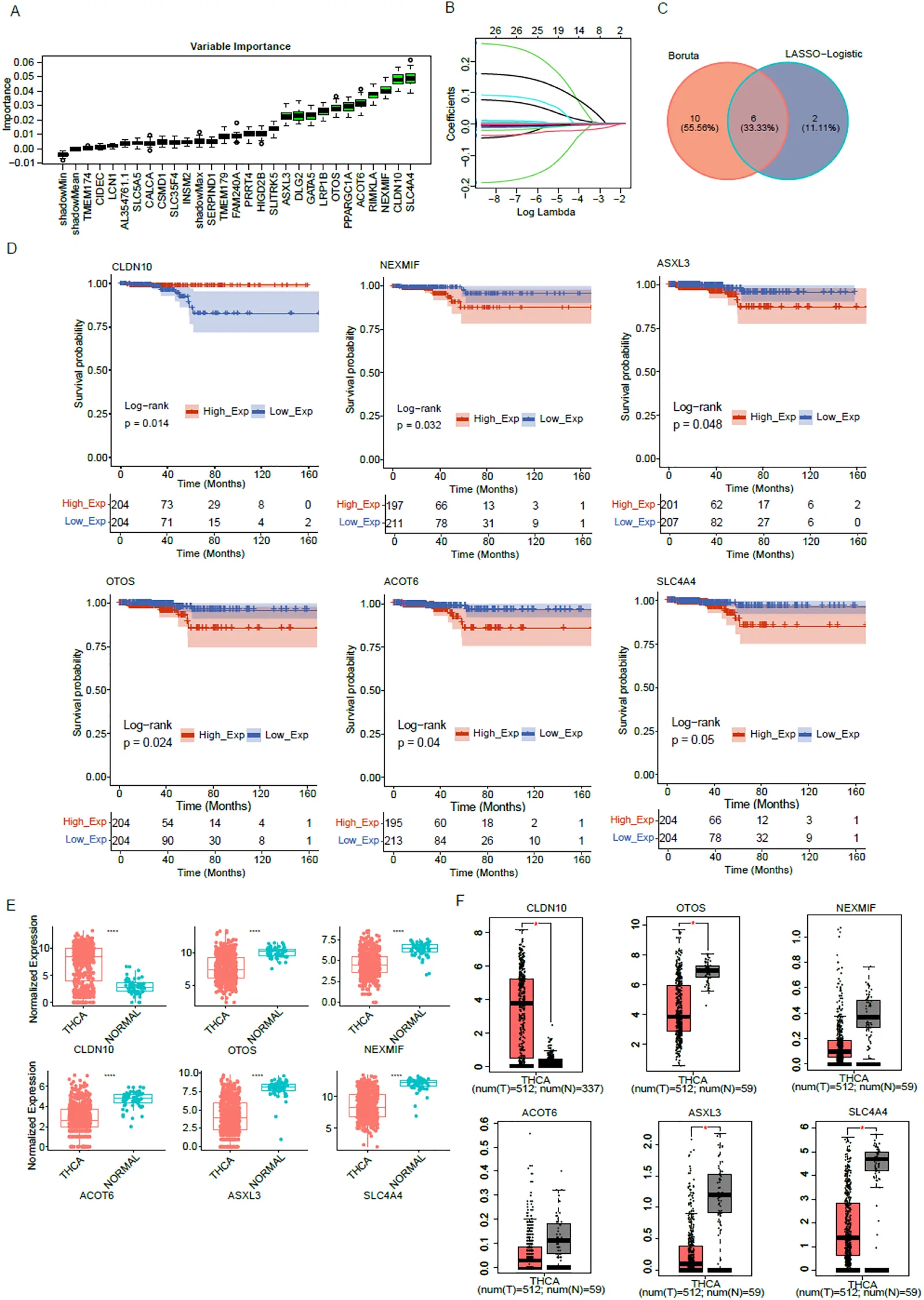
Identification of LNM-associated biomarkers. (A) Box plot showing the importance values for LNM-associated biomarkers. (B) LASSO logistic regression results for screening LNM-associated biomarkers. (C) Venn diagram showing the overlapped LNM-associated biomarkers between Boruta and LASSO results. (D) The overall survival curves for the six biomarkers by classifying THCA patients into two groups. (E) Boxplot showing the expression pattern of six biomarkers from TCGA-THCA patients. (F) The same as (E) but for the GEPIA analysis results.

### LNM-related biomarkers were enriched in signaling pathways associated with immune response and cellular metabolism

Similarly, to elucidate the functions and potential pathways of LNM-related biomarkers, we performed Gene Set Enrichment Analysis (GSEA). Notably, *CLDN10, OTOS, ACOT6, SLC4A4* were co-enriched in allograft rejection, interferon gamma response, and inflammatory response pathway that were associated with immune response.^27^^,^^28^ Additionally, *CLDN10, ACOT6,* and *SLC4A4* were co-enriched in “epithelial mesenchymal transition pathway”, and *OTOS, NEXMIF*, and *ACOT6* were co-enriched in “oxidative phosphorylation pathway”, and *OTOS, ASXL3*, and *SLC4A4* were co-enriched in “interferon alpha response pathway” (Fig. 6A‒F). Collectively, these results underscore the pivotal roles of LNM-related biomarkers in mediating both immune response and cellular metabolism.

**Fig. 6:**
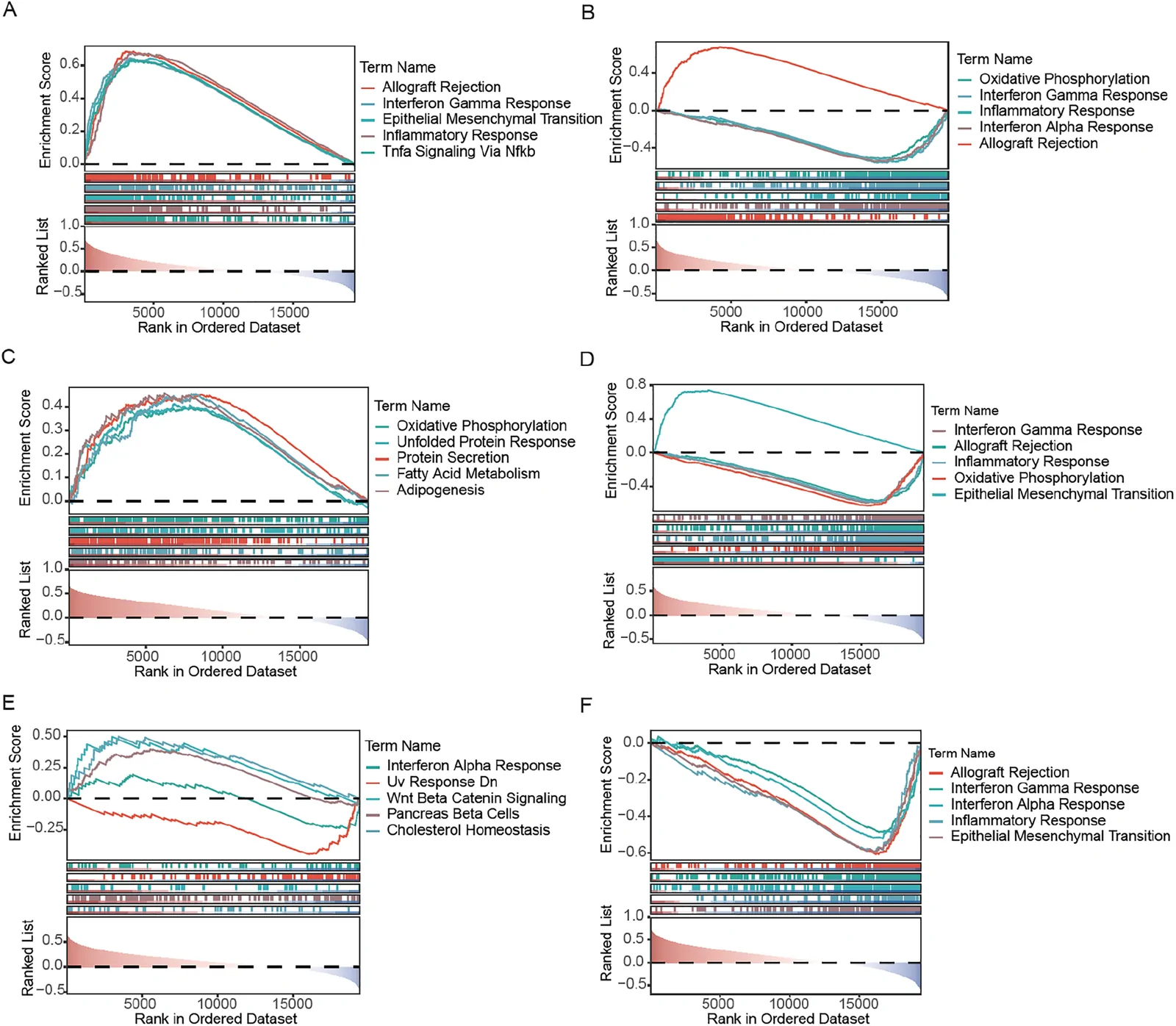
Line plot showing the GSEA results for the correlated genes with the six LNM-associated biomarkers.

### LNM-related biomarkers significantly associated with differential immune cells

Building on enrichment analysis results implicating LNM-related biomarkers in immune pathways, we investigated the role of immune microenvironment in THCA. Using the TCGA-THCA dataset, we performed immune infiltration analysis between THCA and control samples as well as between LNM and NLNM samples to assess abundance of immune cells. Fig. 7A illustrated the proportion of 22 immune cells in each sample within TCGA-THCA dataset. Correlation analysis between the six LNM-related biomarkers and 21 differential immune cells (except for gamma delta T-cells) revealed that *CLDN10* exhibited the strongest positive correlation with resting dendritic cells (*R* = 0.47 and adjusted p < 0.01), while *SLC4A4* showed the strongest negative correlation (*R* = −0.29 and adjusted p < 0.01) (Fig. 7B). Comparing THCA with control samples, we observed significantly elevated proportion of CD8^+^ T-cells, resting memory CD4^+^ T-cells, activated memory CD4^+^ T-cells, T-follicular helper cells, regulatory T-cells, activated Natural Killer (NK) cells, M0 macrophages, M1 macrophages, activated Mast cells, M2 macrophages, resting dendritic cells, activated dendritic cells, resting Mast cells, and activated Mast cells (p < 0.05). In contrast, gamma delta T-cells and eosinophils were significantly reduced (p < 0.001) (Fig. 7C). Similarly, LNM samples showed increased M0 macrophages and resting/activated dendritic cells compared with NLNM samples (p < 0.01), whereas the proportions of monocytes and eosinophil were significantly lower (p < 0.05) (Fig. 7D). Importantly, M0 Macrophages, resting dendritic cells, activated dendritic cells, and eosinophils showed consistent differential patterns across both comparisons (p < 0.05). The results suggest that these significant differences immune cells might influence development of THCA and that LNM-related biomarkers may serve as critical indicators of immune response status in THCA patients.

**Fig. 7:**
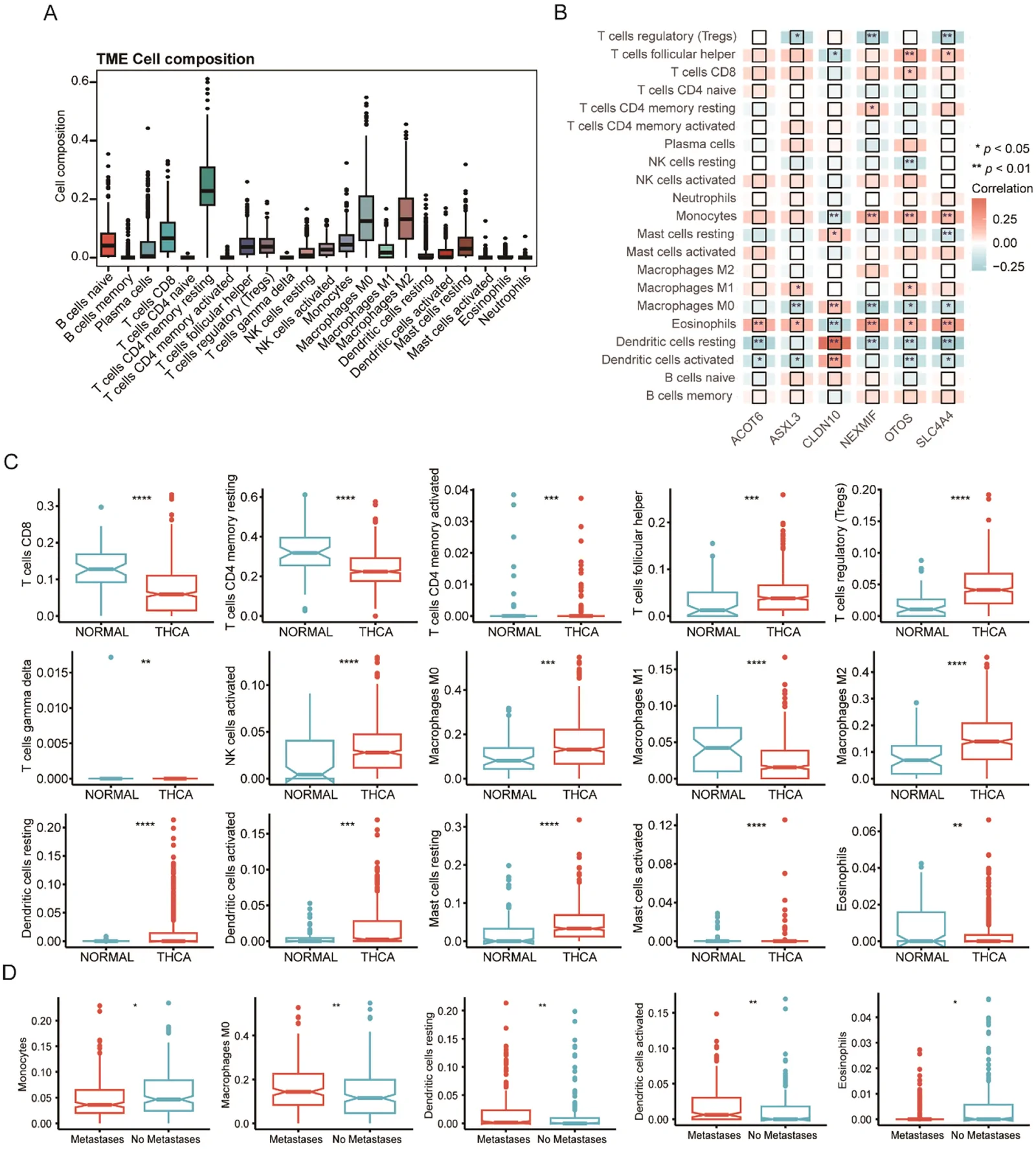
The association analysis between LNM-associated biomarkers and immune cells. (A) Box plot showing the composition of 22 immune cell types. (B) The correlation analysis between 21 immune cells and six LNM-associated biomarkers. (C) Boxplot showing the difference of immune cells between THCA and normal samples. (D) Boxplot showing the difference of immune cells between LNM and NLNM samples.

### Integrative ceRNA network analysis of LNM-related biomarkers

To elucidate the post-transcriptional regulatory network of LNM-related biomarkers, we constructed a ceRNA network analysis. The resulting ceRNA network comprised 14 miRNAs that computationally regulate the six mRNA biomarkers and four lncRNAs, revealing intricate competing interactions. For instance, hsa-miR-149–5p was predicted to potentially target *CKDN10, OTOS, SLC4A4, MALAT1, XIST*, and *NEAT1*. While hsa-miR-15a-5p potentially regulates *SLC4A4, ASXL3, NEXMIF*, and *NEAT1* (Fig. 8). The results indicate the potential regulatory miRNA and lncRNA factors for LNM-related biomarkers in THCA. Meanwhile, future experiments are necessary to validate the potential regulatory network and identify the regulators of these LNM-related biomarkers.

**Fig. 8:**
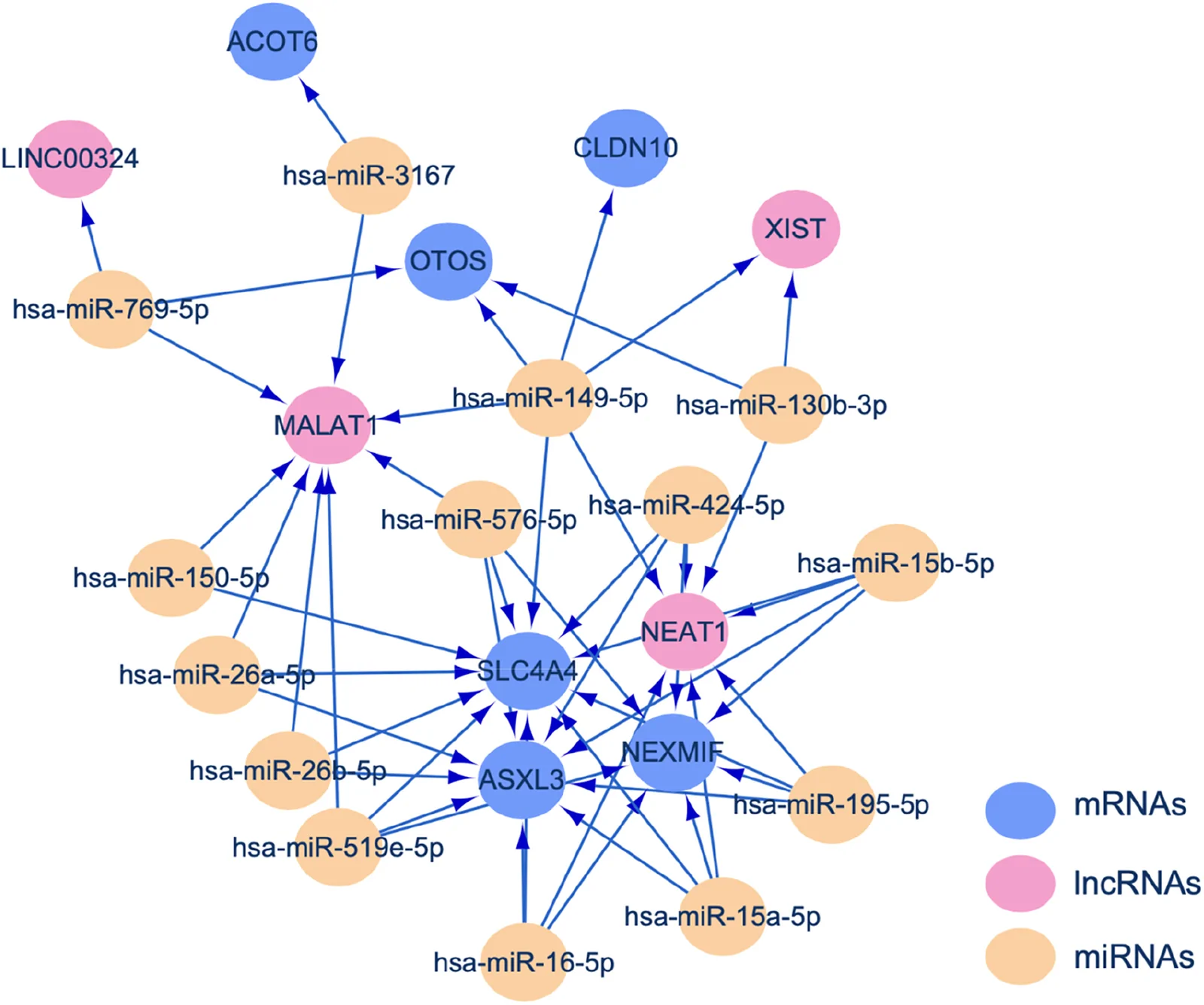
The ceRNA regulatory network among miRNAs, lncRNAs, and mRNAs detected in this study.

## Discussion

Thyroid Carcinoma (THCA) is one of the most commonly diagnosed malignancies worldwide and significantly impacts patient quality of life and survival.^2^ More importantly, the LNM state emerges as a critical determinant of THCA progression and a major predictor of poor outcomes.^7^^,^^29^ Thus, early diagnosis of LNM is essential for optimizing treatment strategies. In this study, we identified both clinical factors and a six-gene biomarker panel that were associated with LNM and survival time of THCA patients. These biomarker genes were enriched in immune response and metabolism pathways, highlighting their potential as novel diagnostic indicators and therapeutic targets for LNM in THCA management.

In this study, we first used clinical information from the SEER database to demonstrate that LNM can play important roles in the development of THCA as LNM group had worse prognosis result.^30^ Consistent results were obtained using both Overall Survival (OS) and Cancer-Specific Survival (CSS) endpoints. In parallel, we developed two nomograms with strong potential for clinical application, which may provide a practical risk-scoring framework for clinical applications.^31^ Subsequently, we later explored genes related to LNM in the TCGA database. Integrating the published studies and our analysis results, we identified six LNM-related biomarker genes that can affect the survival time of THCA patients, including *CLDN10, OTOS, NEXMIF, ACOT6, ASXL3*, and *SLC4A4*. One previous study has demonstrated clinical value of CLDN10 in discriminating tumors from benign thyroid lesions as well as its association with LNM.^32^ The genetic mutations of *ASXL3* have been identified in sporadic parathyroid adenomas, supporting its diagnostic potential.^33^ Decreased *SLC4A4* expression in our THCA cohort corroborates previous reports linking its downregulation to invasion and metastasis in papillary thyroid carcinoma.^34^ While the remining biomarkers have not been extensively studied in the thyroid biology literature, our findings suggest that they may serve as novel targets for LNM diagnosis of LNM in THCA by clinical examination and their functions can be investigated by in vitro cellular experiments to clarify whether they are drivers or passengers in LNM of THCA. For these biomarkers, clinical kits can be conducted to quickly test their expression levels in blood. Immunostaining can also be performed on the tissue samples to predict the probability of LNM for THCA patients. At the same time, future clinical projects are necessary to assess their performance in LNM prediction and compare it to existing tools.

Functional enrichment pathway analysis for the identified biomarkers revealed that they were intimately involved in immune response and metabolism, including allograft rejection, interferon gamma response, and inflammatory signaling pathways. Allograft rejection mobilizes both innate and adaptive immunity, mechanisms increasingly implicated in cancer progression.^35^ Inflammatory response can be triggered by oncoproteins and has been extensively investigated in THCA.^36^ Meanwhile, the THCA microenvironment is densely infiltrated by immune cells that directly interact with epithelial cancer cells and have a profound effect on the growth and metastasis of cancer cells.^37^ Our findings suggest that the identified biomarker may facilitate LNM through immune-inflammatory crosstalk, consistent with recent reports.^38^

Beyond the molecular and immune landscape, our findings must be considered within the evolving clinical framework of thyroid nodule risk stratification. Since its inception following the 2007 National Cancer Institute State of the Science Conference, The Bethesda System for Reporting Thyroid Cytopathology (TBSRTC) has been instrumental in standardizing thyroid FNA reporting and guiding clinical management through its six-category system.^39^ However, Category III ‒ Atypia of Undetermined Significance or Follicular Lesion of Undetermined Significance (AUS/FLUS) ‒ has remained the most contentious 'gray zone' due to its inherent heterogeneity and the variable Risk Of Malignancy (ROM) it conveys.^40^ Recent scholarly discourse has highlighted the crucial need for subcategorizing Category III to refine risk stratification and clinical decision-making.^41^ In line with these discussions, the latest 2023 TBSRTC, third edition has officially adopted a two-tiered subdivision of Category III: AUS-nuclear atypia (AUS-NA) and AUS-other.^42^^,^^43^ This formalization resolves a long-standing debate and provides a more nuanced framework for assessing ROM in indeterminate nodules. The six candidate biomarkers identified in our study (CLDN10, OTOS, NEXMIF, ACOT6, ASXL3, and SLC4A4) could, if validated, complement these cytological and imaging-based risk stratification systems. For instance, integrating molecular biomarkers with refined TBSRTC categories may enhance the prediction of LNM and overall prognosis in THCA patients. Furthermore, the potential interplay between thyroid dysfunction, hormonal status, and immune regulation, as explored in the context of melatonin and menopause,^44^ underscores the need for a multidisciplinary approach to thyroid nodule management.

Immune cell infiltration analysis further revealed significant perturbations in the THCA microenvironment, with M0 macrophages, resting dendritic cells, activated Dendritic Cells (DCs), and eosinophils showing the most pronounced alterations. These observations echo previous studies highlighting the prognostic significance of tumor-infiltrating immune populations in THCA.^45^^,^^46^ Tumor-associated macrophages accumulation correlates with metastasis and reduced survival in advanced THCA patients.^47^ Specifically, rested and activated DCs, which showed the strongest correlations with our biomarkers (particularly *CLDN10* and *SLC4A4*), have been successfully used in therapeutic vaccination strategies for medullary thyroid carcinoma.^48^ Besides THCA, DCs also involve in the crucial immune regulation of early and chronic phases of various autoimmunity types in the thyroid gland,^49^ indicating the therapeutic and predicting roles of DCs in THCA. The expression pattern and functions of CLDN10 and SLC4A4 have been discussed above. The results suggest that CLDN10 and SLC4A4 may regulate THCA by modulating the activation of DCs. Meanwhile, further validating experiments, such as protein-level confirmation by Western blot, functional investigation for key proteins, and clinical verification, should be conducted to verify the LNM-related biomarkers in future.

The ceRNA network analysis uncovered a complex regulatory architecture wherein 14 miRNAs and four lncRNAs (MALAT1, NEAT1, XIST) potentially govern biomarker expression. Among this regulatory network, three famous lncRNAs, including *MALAT1, NEAT1*, and *XIST*, are established oncogenic regulators in THCA,^50^, ^51^, ^52^ and identified as potential regulators on five biomarker genes. At the same time, more miRNAs were identified with potential regulation on the expression of biomarker genes. For example, has-miR-149–5p has been reported to influence the development of multiple cancer types.^53^^,^^54^ This network provides a mechanistic framework for biomarker regulation, though experimental verification is required to establish causality.

At last, our study also has several limitations. The identified hub genes lack experimental validation at the protein and functional levels, including the multiple testing corrections. The six identified biomarkers are not well-studied in thyroid biology, although they were highly expressed in LNM samples of THCA, indicating that further experiments are required to elucidate their functions in THCA and thyroid cancer cell lines. The six biomarkers should also be compared with the well-established thyroid cancer markers, including BRAF, RAS, and TERT to increase their clinical value. Due to the relatively small TCGA cohort (n = 408), for future extension, more samples (at least one thousand) from multiple independent cohorts and better prediction algorithm should be conducted to validate the biomarkers and their clinical value. Experimental validation, including luciferase reporter assays, qPCR, and Western blotting, is necessary to confirm functional regulation of ceRNA network. Finally, immune cell proportions derived from bulk transcriptomics should be confirmed through single-cell RNA sequencing for higher cellular resolution.

## Conclusion

In summary, we conducted an extensive exploration for the biomarkers associated with LNM of THCA. The identified biomarkers require further validated using additional clinical samples, and their regulatory mechanisms require validation through cellular and animal experiments in future studies. The identified biomarkers were significantly enriched in immune and inflammatory pathways, implying their potential function in immune therapy. Meanwhile, the identified LNM-associated biomarkers have not been extensively investigated in THCA, indicating that they can serve as signatures and laid a new perspective for diagnosis, prognosis, and treatment of THCA in future.

## Abbreviations

AUC, Area Under the Curve; CeRNA, Competitive Endogenous RNA; CSS, Cancer-Specific Survival; DCA, Decision Curve Analysis; DEGs, Differentially Expressed Genes; FC, Fold Change; GO, Gene Ontology; GSEA, Gene Set Enrichment Analysis; KEGG, Kyoto Encyclopedia of Genes and Genomes; KM, Kaplan-Meier; LASSO, Least Absolute Selection and Shrinkage Operator; LNM, Lymph Node Metastasis; NLNM, Non-Lymph Node Metastasis; OS, Overall Survival; PPI, Protein-Protein Interaction; ROC, Receiver Operating Characteristic; SD, Standard Deviation; SEER, Surveillance, Epidemiology, and End Results; TCGA, The Cancer Genome Atlas; THCA, Thyroid Carcinoma; UCSC, University of California Santa Cruz.

## Authors’ contributions

All authors listed have made a substantial contribution to this work and approved the manuscript. Conceptualization: Xuejian Hu; Data curation: Dan Ge; Formal analysis: Xuejian Hu and Yan Yang; Methodology and Software: Xuejian Hu and Xiaolan Ma; Visualization: Xuejian Hu and Xiaolan Ma; Funding acquisition: Xuejian Hu; Supervision: Yan Yang; Writing-original draft: Xuejian Hu; Writing-review & editing: Xuejian Hu and Yan Yang.

## Funding

This work was supported by the 10.13039/501100004775Natural Science Foundation of Gansu Province [21JR11RA125 and 24JRRA365].

## Conflicts of interest

The authors declare that they have no known competing financial interests or personal relationships that could influence the work reported in this study.

## Data Availability

The data used and analyzed during the current study are available from the corresponding author on reasonable request.
